# Commentary: Heart Fat Infiltration in Subjects With and Without Coronary Artery Disease

**DOI:** 10.3389/fcvm.2016.00002

**Published:** 2016-02-01

**Authors:** Salvatore Chirumbolo

**Affiliations:** ^1^Department of Medicine, University of Verona, Verona, Italy

**Keywords:** lipid droplet, atrium, adipose tissue, adipocytes, coronary disease

Mazzali et al. showed myocardial steatosis in subjects with coronary artery disease (CAD), describing a significant increase in lipid droplets (LDs) and metabolically active adipocytes interspersed among cardiomyocytes, which were positively associated with changes in BMI and circulating leptin and negatively with adiponectin (1).

The authors used perilipin (PLIN-1) and adipophilin (PLIN-2) markers to detect, respectively, adipocyte or cardiomyocytes LDs and reported that while PLIN-2 was detected in all subjects, about 39% of both CAD and non-CAD patients expressed PLIN-1, showing apparently no relationship in tissue fat distribution between patients and controls. Yet, when intra-cardiomyocyte fat deposits were evaluated, subjects with CAD expressed higher levels of both PLIN-1 and PLIN-2 and higher LD diameter than controls, besides apoptosis and hypoxia markers (1). The observed higher amount of PLIN-1 and PLIN-2, evaluated by immunohistochemistry rather than gene expression, correlated with some metabolic serum markers. While PLIN-1 seems to be associated with larger triacylglycerol LDs in mature adipocytes, PLIN-2 is mainly associated with LDs in non-steroidal adipose tissues (2). LDs are usually collectively grouped in the PAT family, which includes perilipins (P), ADRP (also called adipophilin) (A), Tip-47 (tail-interacting protein of 47 kDa) (T), hence the acronym PAT. They also include S3–12 and OXPAT (also termed MLDP or LSDP5) and recently, respectively, named PLIN1, PLIN2, PLIN3, PLIN4, and PLIN5 (3). The use of PLIN-1 and PLIN-2 is particularly useful in evaluating fat deposits in tissues, although some question still remains, as LD proteins are expressed in a highly complex way during lipidogenesis in the adipose cell (4). In this perspective, some further markers to improve adipocyte detection, such as CIDEC, might be suggested (5–7), in order to better differentiate adipocytes from LDs and also to reduce bias during the immunological detection of perilipin isoforms, as they share a high sequence homology and modification in their N- or C-terminus, depending on their function (4). Unlike other perilipins, PLIN-1 is widely associated with any LD moving from membranes and endoplasmic reticulum to fat droplets during lipid synthesis in any steroidogenic cells and probably may not be a specific marker of isolated white adipocytes (8). Furthermore, the expression of PLIN-2, which the authors associated with LDs in cells different from adipocytes, has also been reported in brown adipocytes (9) and adipocytes from white adipose tissue may express adipophilin (PLIN-2), in particular metabolic conditions (4). Expanding this investigation with further insights may improve the meaning of the reported evidence. For example, perilipin-5 (PLIN-5), which is an important marker in cardiac control of lipolysis (10), is particularly abundant in the heart (11), and as its overexpression could lead to myocardial steatosis, including this marker in future research on myocardial lipidology, particularly in CAD, may improve our knowledge and ability to address this issue (12, 13).

Isolated adipocytes within the myocardium are not a true novelty (14), as adipose tissue was described also within myocardial ventricular walls (15) and recently confirmed in atrial dysfunction (16). Further research in this field should address the origin of these adipocytes, e.g., one could investigate the presence of fibroblast-like preadipocytes in the atrium, which may add further insights on the issue (17). The origin of these adipocytes is fundamental to comprehend their role in CAD. Epicardial adipose tissue (EAT) in specimens may cause possible bias, as adipocytes observed within the atrium may derive from epicardium or even a tissue-related mesenchymal transdifferentiation mechanism (1). Interestingly, pericytes may be sources of preadipocytes, likewise endothelial cells in the adipose tissue, and represent a possible source of the observed adipocytes (18, 19). Once highlighted the origin of interspersed adipocytes, one could question, which is the possible role of these cells, namely if they even play a major role in some compensatory mechanism triggered by myocardial tissue to prevent serious damage due to CAD. The recently reported evidence that at least in skeletal muscle, the increase in PLIN-2 correlated with an improvement in insulin sensitivity in diabetic subjects is intriguing (20). Despite the observation of apoptotic and stress response, signals associated with hypoxia and mitochondrial impairment and dysfunction lead to LDs formation as a positive response to cellular stress (21). In this context, observed adipocytes, which would deserve further clarification about their nature, may shed a light on a possible role in the heart reaction to cardiovascular damage.

PLIN-1 is expressed also by brown adipocytes (8, 22). Further investigation on the main brown adipocyte marker, the uncoupling protein-1 (UCP-1), should differentiate the nature of the observed adipocytes (23, 24). This fact may be important to clarify the role of these cells in the myocardial tissue of CAD patients. Following a suggestion about the possible existence of adipocytes from adult stem cells (ASCs), brown adipose tissue (BAT) is an interesting precursor of cardiomyocytes, as subepicardial fat embryologically derives from BAT and because in aged hearts, a protective mechanism of differentiation of subepicardial stem cells into BAT was recently described (23). The use of UCP-1, to elucidate the nature of interspersed adipocytes, may support the hypothesis that adipocytes serve as an important source to compensate myocardial damage in CAD patients. Adipocytes within myocardial tissue may even have a protective, damage-repairing role, a suggestion supported by the recent evidence that adipose tissue-derived stem cells exert paracrine actions that may have therapeutic effect on myocardial dysfunction (25).

While the authors showed a greater myocardial steatosis in subjects with CAD compared with controls, further research should deepen the role of interspersed adipocytes, their origin, and cytological nature. Suggestions coming from animal models and further experimental research will add insights to the nature of this study, highlighting the mechanism of the mesenchymal source and the role of these cells in the atrium.

## Author Contributions

SC: planned, conceived, wrote, and submitted the whole manuscript.

## Conflict of Interest Statement

The author declares that the research was conducted in the absence of any commercial or financial relationships that could be construed as a potential conflict of interest.
